# A novel RNA molecular signature for activation of 2′-5′ oligoadenylate synthetase-1

**DOI:** 10.1093/nar/gku1289

**Published:** 2014-12-04

**Authors:** Virginia K. Vachon, Brenda M. Calderon, Graeme L. Conn

**Affiliations:** 1 Department of Biochemistry, Emory University School of Medicine, Atlanta, GA 30322, USA; 2 Microbiology and Molecular Genetics (MMG) Program, Graduate Division of Biological and Biomedical Sciences, Emory University School of Medicine, Atlanta, GA 30322, USA; 3 Biochemistry, Cell and Developmental Biology (BCDB) Program, Graduate Division of Biological and Biomedical Sciences, Emory University School of Medicine, Atlanta, GA 30322, USA

## Abstract

Human 2′-5′ oligoadenylate synthetase-1 (OAS1) is central in innate immune system detection of cytoplasmic double-stranded RNA (dsRNA) and promotion of host antiviral responses. However, the molecular signatures that promote OAS1 activation are currently poorly defined. We show that the 3′-end polyuridine sequence of viral and cellular RNA polymerase III non-coding transcripts is critical for their optimal activation of OAS1. Potentiation of OAS1 activity was also observed with a model dsRNA duplex containing an OAS1 activation consensus sequence. We determined that the effect is attributable to a single appended 3′-end residue, is dependent upon its single-stranded nature with strong preference for pyrimidine residues and is mediated by a highly conserved OAS1 residue adjacent to the dsRNA binding surface. These findings represent discovery of a novel signature for OAS1 activation, the 3′-single-stranded pyrimidine (3′-ssPy) motif, with potential functional implications for OAS1 activity in its antiviral and other anti-proliferative roles.

## INTRODUCTION

The cellular innate immune system is the first line of defense against invading pathogens. Innate immunity proteins must detect pathogen-associated molecular patterns (PAMPS) indicative of infection, while still maintaining the stringency required to avoid inadvertent self activation. One potent PAMP is cytosolic double-stranded RNA (dsRNA), produced as a consequence of viral replication (1). This foreign nucleic acid is detected by cellular dsRNA sensors, such as the 2′-5′ oligoadenylate synthetase (OAS) family of enzymes, each with distinct but overlapping specificities, which elicit host antiviral responses (2–6). Activated OAS1 synthesizes 2′-5′-linked oligoadenylate (A(2′-5′A)_n_) second messengers that then activate their only known target, the latent cellular ribonuclease RNase L, to shut down synthesis of host and viral proteins, thereby preventing viral replication.

The molecular evolution of this host system for efficient detection of viruses is countered by co-evolution of viral strategies to thwart the effects of these antiviral proteins. In the case of OAS, these viral strategies include direct inhibition, sequestration of dsRNA, 2′-5′ phosphodiesterases and production of 2′-5′ analogs (7–10). This variety and abundance of viral countermeasures highlights the central importance of the OAS/RNase L antiviral pathway. Indeed, viral mRNA susceptibility to RNase L cleavage is correlated with virus fitness (11), and susceptibility to viruses such as West Nile Virus and Dengue Virus can be mapped to polymorphisms in OAS isoform 1 (OAS1) (12,13). In spite of this growing body of evidence that viral evasion of OAS is important for effective replication of a range of viruses, many details of molecular control of this enzyme family remain unknown.

OAS1 is activated by cytosolic dsRNA with a minimum length of 17 base pairs (bp) and is strongly dependent on direct interaction with a single guanosine residue near the 3′-end of a previously reported activation consensus motif (WWN_9_WG; where W is A or U) (14,15). The recent X-ray crystal structure of human OAS1 bound to a model 18-bp dsRNA duplex revealed that dsRNA binding allosterically drives a functionally essential structural reorganization within OAS1 that narrows the adenosine triphosphate (ATP)-binding cleft and repositions a catalytic residue to complete its active site (14). Although this structure allowed for some rationalization of RNA features currently known to activate OAS1 (15,16), the range of RNA structural and sequence motifs that drive OAS1 activation and the contexts in which such sequences function are not well defined.

The non-coding adenoviral associated RNA-I (VA RNA_I_) accumulates to high levels in late stages of infection, and is critical for efficient adenoviral replication (17). In contrast to its established potent inhibition of dsRNA-activated kinase (PKR) (18),VA RNA_I_ activates OAS1 but may be transformed into a pseudoinhibitor by the action of the cellular RNase Dicer (19–21). We therefore selected VA RNA_I_ as a model system to expand our understanding of the RNA sequences and disparate structural features that regulate OAS1 activity. Our initial analyses of VA RNA_I_-mediated activation of OAS1 uncovered an unexpected RNA molecular signature for OAS1 activation, which we term ‘3′-ssPy’. Here, we describe the discovery and mechanistic characterization of this novel potential PAMP recognized by OAS1. Our findings suggest a potential mechanism for innate immune signal amplification by RNase L products and offer new insight into host–pathogen interaction and the emerging anti-proliferative cellular roles of the OAS/RNase L pathway.

## MATERIALS AND METHODS

### RNA *in vitro* transcription

VA RNA_I_, EBER-1 RNA and nc886 RNA were *in vitro* transcribed from linearized plasmid DNA templates using T7 RNA polymerase as described previously (22–24). All *in vitro* transcribed RNAs, except nc886 RNA, were purified by denaturing polyacrylamide gel electrophoresis (PAGE) and recovered as previously described (25). nc886 RNA was annealed in 0.5xTBE buffer containing 10-mM KCl and purified by preparative native PAGE. nc886 bands were identified by UV shadowing, excised from the gel, eluted by ‘crush and soak’ and recovered by ethanol precipitation.

To generate 18-bp dsRNA duplexes, unmodified reverse strand and each forward strand were synthesized using established procedures. Unmodified reverse strand and each forward strand with zero, one, two, four or eight uridines at the 3′-end were chemically synthesized (Integrated DNA Technologies) and used without further purification. Forward strands with modified 3′-end uridine residues were chemically synthesized and High Performance Liquid Chromatography (HPLC) purified (Dharmacon). Reverse strand with 5′-triphosphate was *in vitro* transcribed using chemically synthesized DNA template and primer under conditions optimized for short transcripts (26), and purified by denaturing PAGE. To generate duplexes, individual strands were mixed in equimolar concentration and annealed by heating to 85°C and slowly cooling to 20°C.

### OAS1 expression and purification

Human OAS1 (p41/E16 isoform) was expressed in *E. coli* BL21(DE3) as a SUMO-OAS1 fusion protein from vector pE-SUMOpro (LifeSensors). Cells for OAS1 expression were grown in Lysogeny Broth (LB) medium at 37°C to an OD_600_ of 0.4, protein expression induced with 0.1-mM isopropyl β-D-1-thiogalactopyranoside and growth continued overnight at 20°C. Cells were lysed by sonication in 50-mM Tris-HCl buffer (pH 8.0) containing 150-mM NaCl, 10-mM imidazole, 1-mM DTT and 10% (v/v) glycerol. Fusion protein was purified by sequential Ni^2+^-affinity and heparin-affinity chromatographies on an ÄKTA Purifier10 system (GE Healthcare). The fusion protein was then dialyzed overnight against SUMO cleavage buffer and cleaved with SUMO protease according to the manufacturer's instructions. Cleavage with SUMO protease leaves a native N-terminus, an important feature of the system as amino acid additions to the N-terminus of OAS1 have been reported to interfere with activity (27).

### OAS1 activity assay using radiolabeled ATP

Polymerization of ATP was observed using α-^32^P-ATP and resolution of 2′,5′-oligoadenylate products by denaturing PAGE. Reactions (10 μl) contained 1 μl of 10-μg/ml OAS1 in 100-mM Tris-HCl, pH7.0, 200-mM NaCl and 10% glycerol; 5 μl of 2 × reaction buffer (40-mM Tris-HCl, pH 7.4, 40-mM magnesium acetate and 5-mM DTT); 1 μl of 100-μM ATP (unless otherwise noted); 0.5 μCi of α-^32^P-ATP (800 Ci/mmol); and either 18-bp dsRNA at final concentrations of 2, 5 or 10 μM, or 50-μg/μl poly(I):poly(C) as activating RNA. Reactions were incubated at 30°C for 3 h then stopped by addition of 3.5 volumes of a 4:25 (v/v) mixture of 1-M Tris-HCl and gel loading solution (85% formamide, 0.5×TBE pH 8.0 and 50-mM ethylenediaminetetraacetic acid (EDTA)) and heating to 95°C for 5 min. Reaction products were resolved on a 20% polyacrylamide, 50% (w/v) urea, 1× TBE PAGE sequencing gel run at 55 W for 1.5 h. Gels were soaked (water; 10 min), fixed (40:10:10% methanol:acetic acid:glycerol; 15 min), dried and then imaged using a Typhoon Trio phosphorimager (GE Healthcare). Band intensities were quantified using ImageQuant software, normalized to the 2′-5′ oligoadenylate product of the highest intensity on the gel and measured values plotted for comparison using Prism 6 (GraphPad).

### Chromogenic assay of OAS1 activity

PPi produced as a result of 2′,5′-oligoadenylate synthesis was monitored in triplicate using a chromogenic assay adapted from previously established methods for measurement of OAS1 activity (28). OAS1 (300 nM) was incubated with 20-μg/ml poly(I):poly(C), 300-nM non-coding RNA (VA RNA_I_, EBER-1 or nc886 RNA) or 1-μM 18-bp dsRNA (unless otherwise stated). These established optimal conditions for OAS1 allowed direct comparisons of activity for RNA and protein sequence variants. Detailed kinetic experiments (measurement of *V*_max_ and RNA *K*_app_) were performed similarly but using RNA in the range 0.1–3 μM, and OAS1 at 300 nM.

OAS1 (300 nM) was incubated with RNA in the presence of 20-mM Tris-HCl, 7-mM MgCl_2_, 1-mM DTT and 1.5-mM ATP in 150-μl total volume at 37°C. Aliquots (10 μl) were removed and immediately quenched by adding to 250-mM EDTA (2.5 μl), pre-loaded into the wells of a 96-well plate. At completion of the time course, molybdate reagent (10 μl, 2.5% ammonium molybdate in 2.5 M H_2_SO_4_), β-mercaptoethanol (10 μl, 0.5 M) and Eikonogen reagent (4 μl, 13-mM 1-amino-2-napthol-4-sulfonic acid, 25-mM sodium sulfite and 963-mM sodium meta-bisulfite dissolved in hot water and filter sterilized) were added to each well and the final volume brought to 100 μl with water. Absorbance at 580 nm was measured using a Synergy4 plate reader (BioTek) and readings converted to PPi produced determined by comparison with PPi standards (28).

While our OAS1 protein showed minimal prep-to-prep variability (Supplementary Figure S1B), we did observe a time-dependent decrease in activity after purification. Because this time-dependent decrease in activity resulted in a proportional change in OAS1 activity for all test RNAs and poly(I):(C), comparisons between experimental sets were made after normalizing to the activity of OAS1 in response to an appropriate control RNA used in each set of experiments. Where data are presented as nmols PPi produced, these data were collected in triplicate using a single OAS1 prep.

For kinetic analyses of OAS1 activation, measurements were made at 5-, 10- and 20-min time points as described above, and linear regression analysis used to obtain the nmols PPi produced/minute for each RNA concentration. Values were plotted using Prism 6 (GraphPad), and curves fit using non-linear regression to obtain *V*_max_ and *K*_app_ values using the Michaelis–Menten model equation *Y* = (*V*_max_*X*)/(*K*_app_ + *X*). For data comparing the effect of the 3′-ssPy motif on wild-type versus mutant OAS1 proteins, statistical analysis was by one-way ANOVA with significance assessed by Sidak's multiple comparisons test in Prism 6 (GraphPad).

## RESULTS

### OAS1 activation is strongly potentiated by the single-stranded 3′-end of VA RNA_I_

**Figure 1.**
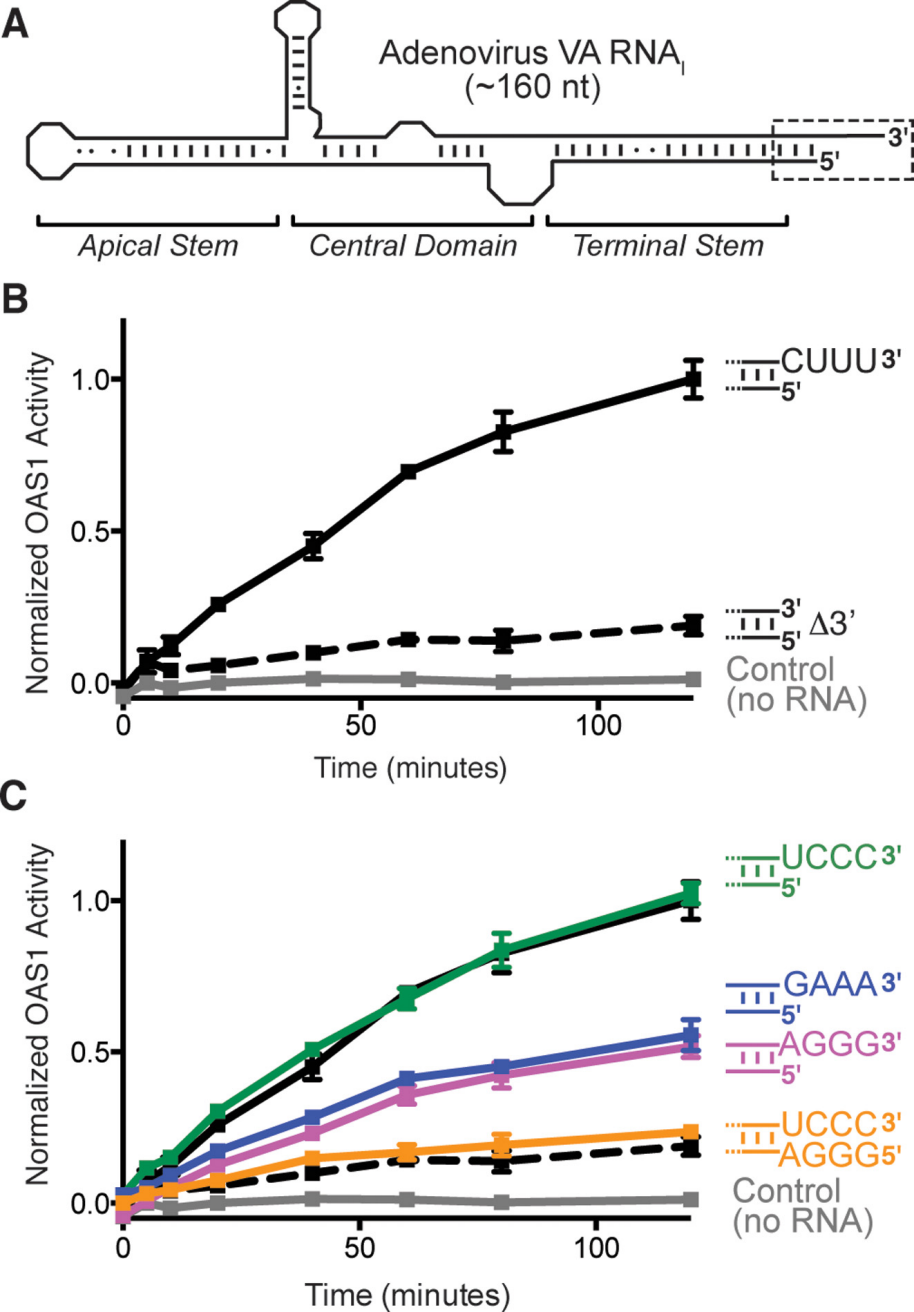
Activation of OAS1 by adenovirus VA RNA_I_ requires its single-stranded pyrimidine-rich 3′-end. (**A**) VA RNA_I_ secondary structure and domain organization. The Terminal Stem 3′-end contains a single-stranded sequence (CUUU-3′; dashed box and shown as sequence in subsequent panels). (**B**) Chromogenic assay of OAS1 activity shows that deletion of the wild-type 3′-end single-stranded sequence (Δ3′; dashed line) dramatically reduces OAS activation by full-length VA RNA_I_ (CUUU-3′; solid line). (**C**) Alternate pyrimidine-rich (UCCC-3′) or purine-rich (AGGG-3′ or GAAA-3′) single-stranded 3′-ends promote full and partial activation of OAS1, respectively. Addition of a complementary 5′-end extension to fully base pair the UCCC-3′ sequence (5′-AGGG/UCCC-3′) attenuates OAS1 activation to the same extent as when the 3′-end single-stranded sequence is absent. The data in panels (B) and (C) are normalized to wild-type VA RNA_I_ (CUUU-3′).

We created a bacterial expression system to produce recombinant human OAS1 as an N-terminal SUMO protein fusion. Cleavage of the fusion protein with SUMO protease yielded the authentic OAS1 N-terminus and protein which displayed robust activation by poly(I).poly(C) dsRNA in two complementary assays with low prep-to-prep variability (Supplementary Figure S1). In contrast, our initial experiments failed to recapitulate previous observations of OAS1 activation by VA RNA_I_ (19,21). Our VA RNA_I_ *in vitro* transcript differed from those used previously only by the absence of a single-stranded four nucleotide sequence appended to the 3′-end of VA RNA_I_ (denoted CUUU-3′; Figure 1A and Supplementary Figure S2A) as a consequence of RNA Polymerase III (RNA Pol III) transcription termination *in vivo* (29). This 3′-end single-stranded sequence has no effect on PKR inhibition by VA RNA_I_ and was removed in our construct as we found this simplified purification of the *in vitro*-transcribed RNA (25,30). Remarkably, restoration of the complete wild-type sequence to include the CUUU-3′ sequence dramatically enhanced OAS1 activation (Figure 1B and Supplementary Figure S1).

To understand the unexpected contribution of this sequence to OAS1 activation, we first asked whether the sequence of the single-stranded 3′-end is critical. Three VA RNA_I_ variants were generated in which the single-stranded CUUU-3′ sequence was mutated to alternative pyrimidine-rich (UCCC-3′) or purine-rich sequences (GAAA-3′ or AGGG-3′). VA RNA_I_ containing the alternative pyrimidine-rich sequence (UCCC-3′) activated OAS1 as effectively as wild-type (Figure 1C). In contrast, each of the two purine-rich 3′-end sequences enhanced activation of OAS1 equally, but not to the same extent as either pyrimidine-rich sequence.

To confirm that the stimulatory effect of the 3′-end sequence is dependent upon it being single stranded, we created a further variant of the UCCC-3′ construct with a fully complementary sequence also appended to the 5′ end (5′-GGGA; Figure 1C). The UCCC-3′ sequence was selected, rather than the wild-type 3′-end, as its complement retains the necessary 5′-end nucleotides for initiation of T7 RNA polymerase *in vitro* transcription. The ability of this extended base paired VA RNA_I_ (5′-GGGA/UCCC-3′) to activate OAS1 was attenuated to the same extent as when the 3′-single-stranded sequence was absent (Figure 1C). Thus, the stimulatory effect of the additional 3′-end sequence does not simply arise through extension of the dsRNA length. Collectively, these results demonstrate that maximal activation of OAS1 by VA RNA_I_ is dependent upon its 3′-end being both pyrimidine-rich and single-stranded in nature. This is particularly striking given that OAS1 is a tightly controlled enzyme activated exclusively by dsRNA. We term this novel potentiator of OAS1 activation the 3′-single-stranded pyrimidine (3′-ssPy) motif.

### The 3′-ssPy motif enhances OAS1 activation by other structured non-coding RNAs and a short dsRNA duplex

To determine whether the enhancement of OAS1 activation by the 3′-ssPy motif is a general phenomenon, we examined the requirement for similar sequences in two other structured RNA Pol III transcripts: the non-coding Epstein–Barr virus encoded RNA 1 (EBER-1) and the cellular non-coding RNA 886 (nc886) (Supplementary Figure S2B and C). Like VA RNA_I_, EBER-1 inhibits PKR but activates OAS1 (31). We found that full-length EBER-1 activated OAS1 to the same degree as VA RNA_I_, and deletion of its single-stranded 3′-end RNA Pol III termination signal (UUUU-3′) also dampened its activity (Supplementary Figure S3A). The cellular transcript nc886 is another potent inhibitor of PKR (32), but its activity against OAS1 was not previously tested. We found that nc886 demonstrates a remarkable ability to activate OAS1 that is again attenuated upon deletion of its UUUU-3′ sequence (Supplementary Figure S3B). Together, these results demonstrate that the effect of the 3′-ssPy motif in potentiating OAS1 activity is general among these structurally complex non-coding RNAs.

**Figure 2.**
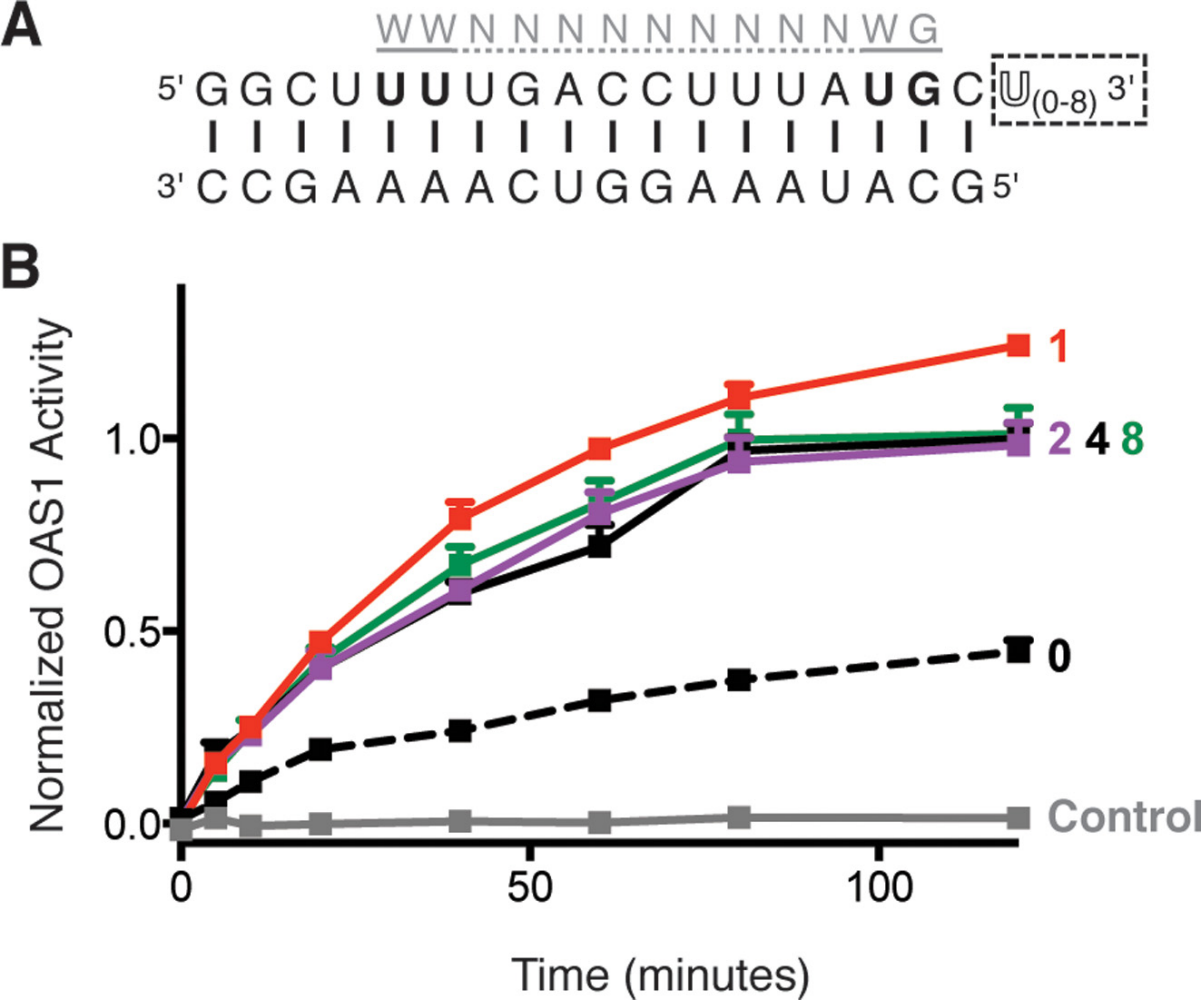
The 3′-ssPy motif potentiates OAS1 activation by a simple dsRNA duplex containing an OAS1 consensus sequence. (**A**) Sequence of the 18-bp dsRNA duplex highlighting the location of the known OAS1 consensus sequence (gray) and additional 3′-end single-stranded uridine residues (3′-ssPy motif; dashed box). (**B**) Analysis of OAS1 activation by 18*-*bp dsRNA duplexes with zero, one, two, four or eight single-stranded 3′-end uridine residues using the chromogenic assay. In both panels data are normalized to the 18-bp dsRNA with four 3′-end single-stranded U residues.

We next asked whether the 3′-ssPy motif could potentiate OAS1 activation by a simple dsRNA using the model 18-bp duplex cocrystallized with OAS1 (14). In this context, addition of a single-stranded UUUU-3′ sequence enhanced OAS1 activation (Figure 2). This pronounced potentiation of OAS1 activity is particularly surprising as the model dsRNA contains a potent activation consensus sequence (15). Thus, the 3′-ssPy motif is effective in potentiating the activity of both non-coding RNAs with complex secondary structures and simple dsRNAs.

Having established that the 3′-ssPy motif is effective in the 18-bp dsRNA duplex, we used this model system to determine the length requirement of the 3′-end single-stranded region for optimal OAS1 activation. We tested activity of the 18-bp dsRNA duplex with one, two, four or eight single-stranded uridine residues (Figure 2B) and found that a single uridine residue appended to the 3′ end of the dsRNA was sufficient to confer the maximum potentiation of OAS1 activity. No further enhancement of OAS1 activation was observed upon extension of the 3′-end single-stranded sequence. Further, the 3′-ssPy motif cannot exert an effect unless it is appended to a double-stranded helical structure, as neither individual strand of the 18-bp duplex RNA, with or without an additional UUUU-3′ sequence, is able to activate OAS1 (Supplementary Figure S4).

### The 3′-ssPy motif increases OAS1 activity but does not specifically alter A(2′-5′A)_n_ product lengths

**Figure 3.**
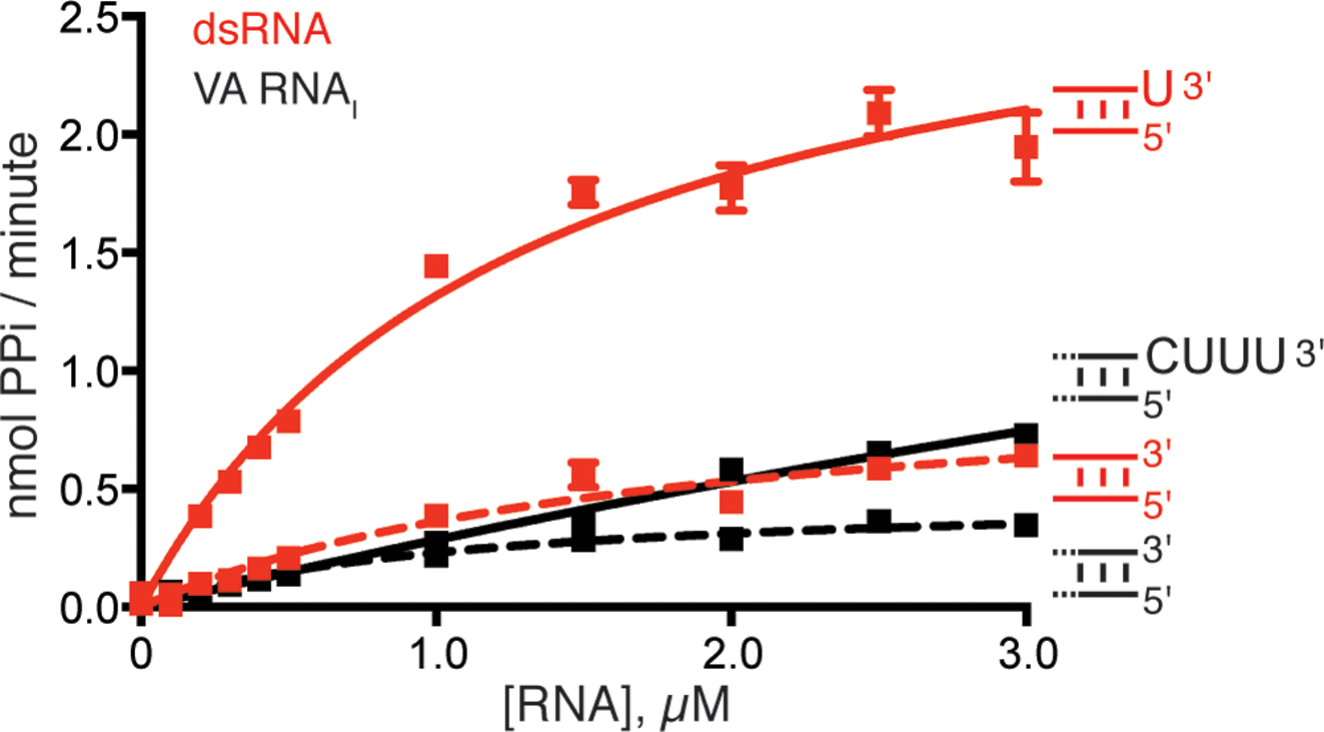
Kinetic analysis of OAS1 activation by RNAs with and without a 3′-ssPy motif. OAS1 activity over a range of VA RNA_I_ and 18-bp dsRNA duplex concentrations both with (solid lines) and without (dashed lines) the 3′-ssPy motif (3′-CUUU and 3′-U, respectively). Data were fit using non-linear regression to obtain the kinetic parameters (*V*_max_ and *K*_app_) shown in Table 1.

To determine the effect of the 3′-ssPy motif on catalysis of A(2′-5′A)_n_ synthesis, we performed enzyme kinetic analyses of OAS1 activity with respect to activator RNA concentration. This analysis was performed for both the 18-bp dsRNA duplex and VA RNA_I_ with and without a 3′-ssPy motif (U-3′ and CUUU-3′, respectively; Figure 3 and Table 1). For the 18-bp dsRNA duplex, the presence of the 3′-ssPy motif increased OAS1 V_max_ by 3-fold but had no apparent effect on RNA affinity (*K*_app_). For VA RNA_I_, both *V*_max_ and *K*_app_ are increased by the presence of the CUUU-3′ sequence by ∼8- and 12-fold, respectively. The 3′-ssPy-mediated increase in OAS1 *V*_max_ with both RNAs suggests that the effect of 3′-ssPy is similar in each context and arises, at least in part, by promoting OAS1 catalytic activity. However, the context-specific influence of the 3′-ssPy motif on OAS1–RNA interaction (*K*_app_) reflects an apparent decrease in RNA affinity that may be due to the fact that the dsRNA duplex has a single optimal binding orientation, whereas VA RNA_I_ contains multiple potential binding sites of differing affinity and capacity to activate OAS1 (see the Discussion section).

**Table 1.** Influence of 3′-ssPy on OAS1 activity

RNA	*V*_max_ (nmol PPi/min)	*K*_app_ (RNA) μM
18-bp dsRNA	1.0 ± 0.2	1.8 ± 0.5
18-bp dsRNA(3′-U)	3.0 ± 0.3	1.3 ± 0.3
VA RNA_I_ (ΔCUUU)	0.48 ± 0.05	1.1 ± 0.2
VA RNA_I_	4.0 ± 1.7	13.1 ± 6.6

**Figure 4.**
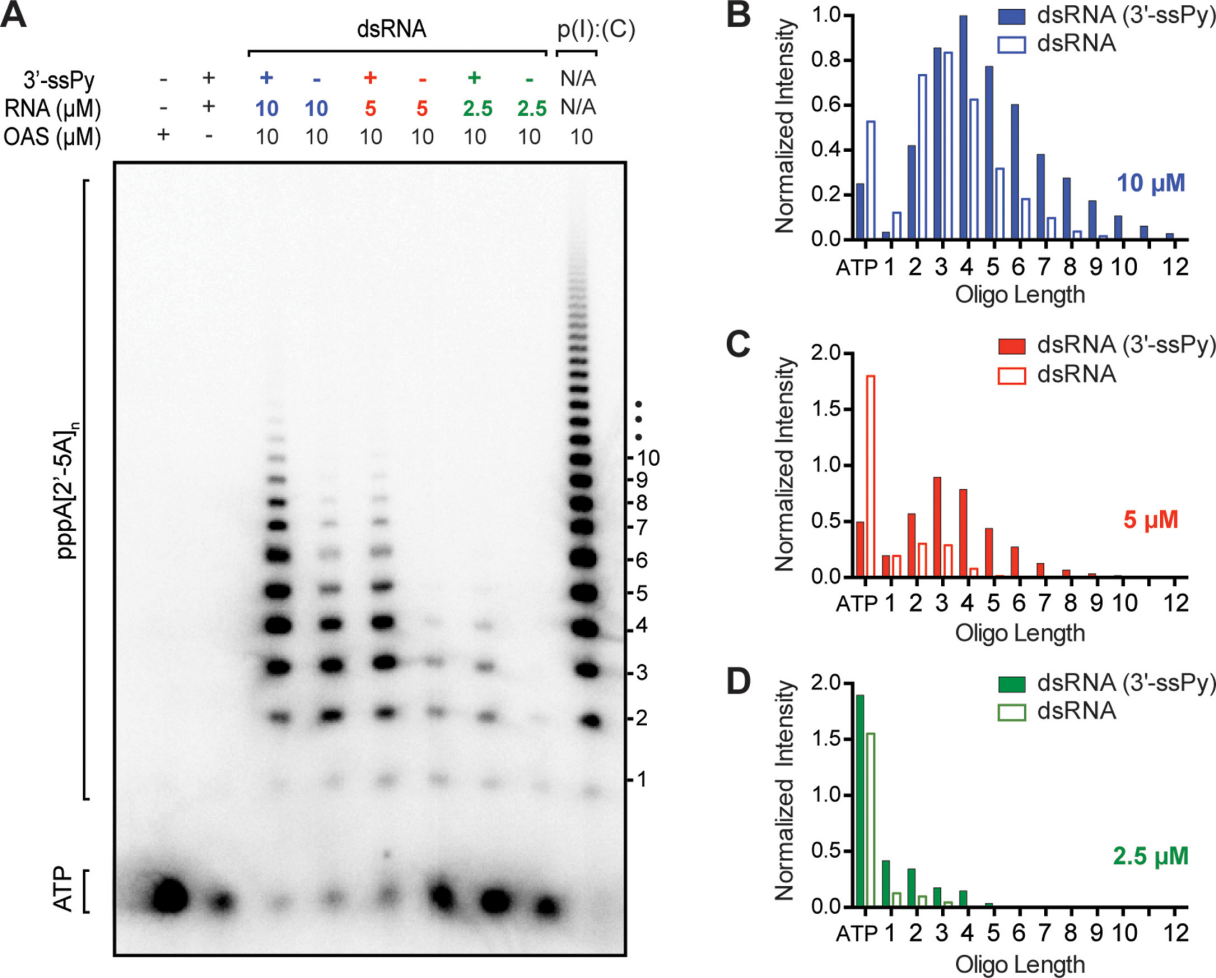
The 3′-ssPy motif causes an increase in OAS1 activity but not an altered accumulation of specific product lengths. (**A**) Phosphorimager analysis of denaturing PAGE showing OAS1 synthesis of 2′-5′ oligoadenylates in the presence of 18-bp dsRNA activator with and without the 3′-ssPy motif at three different concentrations (10, 5 and 2.5 μM). OAS1 activation at a single concentration of the known activator poly(I).poly(C) RNA is shown for comparison. (**B–D**) Quantification of 2′-5′ oligoadenylate product band intensities in the presence of (B) 10-μM, (C) 5-μM and (D) 2.5-μM 18*-*bp dsRNA activator with (solid bars) or without (open bars) the 3′-ssPy motif. All bands were normalized to the most intense product band produced (i.e. *n* = 4 oligoadenylate in the lane third from left, corresponding to 10-μM dsRNA with a 3′-ssPy). Remaining α-^32^P-ATP is shown on the far-left of the x-axis.

Relative abundance of A(2′-5′A)_n_ product length can have significant functional consequences. For example, A(2′-5′A) dimers promote RNase L-mediated ribosomal stop codon readthrough, whereas A(2′-5′A)_n≥2_ oligomers induce its ribonuclease activity (33). The chromogenic assay effectively measures differences in overall OAS1 activity but cannot distinguish inorganic pyrophosphate (PP_i_) produced through synthesis of oligoadenylates of specific lengths. Therefore, to determine whether 3′-ssPy influences the distribution of oligoadenylate products, we used denaturing polyacrylamide electrophoresis (PAGE) to separate the products of OAS1 incubated with three different concentrations of 18-bp dsRNA duplex both with and without a 3′-ssPy motif (Figure 4). We found that the stimulatory effect of the 3′-ssPy motif did not result in accumulation of products of specific length. Instead, similar to the general stimulatory effect of increasing the dsRNA activator concentration, at each individual dsRNA concentration the 3′-ssPy motif resulted in higher overall activity and thus production of more oligoadenylate of greater length. Thus, from the two complementary assays of OAS1 activity, the difference between RNAs with and without 3′-ssPy appears to arise through an overall increase in OAS1 activity.

### Modifications of the 3′-ssPy ribose distinguish chemical features responsible for mediating OAS1 interactions

**Figure 5.**
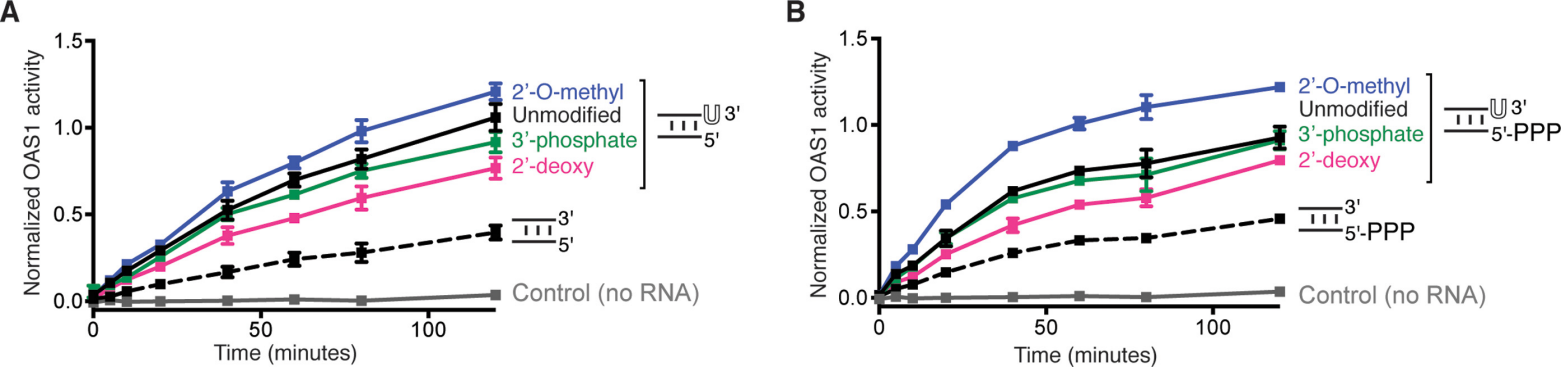
3′-end modifications have minor effects on 3′-ssPy activity and are relatively unaltered by 5′-ppp on the reverse strand of 18-bp RNA. (**A**) Assays of OAS1 activation by dsRNA duplexes with chemical modifications to the 3′-ssPy motif ribose group: 2′-O-methyl, 2′-deoxyribose and 3′-phosphate. dsRNA with unmodified 3′-ssPy (one single-stranded 3′-end uridine) and without a 3′-ssPy motif are shown for comparison. None of the modifications reduced activity to the level of the dsRNA lacking a 3′-ssPy motif but subtle changes in activation suggest an influence of the ribose sugar pucker on 3′-ssPy motif activity. (**B**) As panel (A) but for dsRNA duplexes with a 5′-triphosphate group on the complementary strand. The 5′-triphosphate group did not alter relative effects of the modifications on 3′-ssPy motif potency but does appear to modestly enhance activation by the 3′-ssPy motif with 2′-O-methyl ribose modification. In all panels data are normalized to the 18-bp dsRNA with the unmodified 3′-ssPy motif.

Innate immune proteins that sense dsRNA often depend on interactions with the ribose 2′-OH within the structurally open dsRNA minor groove. For example, 2′-OH-mediated binding is the primary determinant of dsRNA specificity for PKR (34) and introduction of 2′-O-methyl groups into the 18-bp dsRNA duplex attenuate OAS1 activation (15). To determine whether the 3′-ssPy motif 2′-OH or other feature of ribose is critical for its activity, we prepared 18-bp dsRNA duplexes with one of the three chemical modifications in a single 3′-end single-stranded uridine residue: 2′-O-methyl, 2′-deoxyribose and 3′-phosphate. We compared the ability of the unmodified and modified dsRNAs to activate OAS1 and found that the effect of the 3′-ssPy motif was increased to a small degree by the 2′-O-methyl group, and only minimally attenuated by 2′-deoxyribose (Figure 5A). However, none of the modifications reduced activity to the level of the duplex lacking the 3′-ssPy.

The presence of a 5′-triphosphate group may also enhance OAS1 activation in some contexts (21). Therefore, each dsRNA duplex was additionally tested with a 5′-end triphosphate group on the complementary strand. The 5′-triphosphate group had no effect on OAS1 activation by the 18-bp dsRNA duplex with or without a 3′-ssPy motif and also did not alter the relative effects of the 3′-ssPy modifications (Figure 5B and Supplementary Figure S5). However, the modest enhancement of OAS1 activation mediated by the 2′-O-methyl-modified 3′-ssPy motif was more pronounced in the presence of a 5′-triphosphate on the complementary strand. Collectively, these results suggest that 3′-ssPy action is not mediated by direct interaction of OAS1 with its ribose 2′-OH group nor is the 3′-phosphate group required. Instead, 3′-ssPy action appears to be more subtly influenced by the ribose conformation as modifications likely to promote A-form (C3′-*endo*; 2′-methyl) or B-form (C2′-*endo*; 2′-deoxy) ribose sugar pucker modestly enhance or attenuate 3′-ssPy motif potency, respectively. Additionally, these results indicate that while viral RNAs are not known to be extensively 2′-O-methylated, such modification of viral or cellular RNAs would not impact activation of OAS1 by the 3′-ssPy motif.

### Mutagenesis of OAS1 identifies a critical residue that mediates the effect of the 3′ssPy motif

Our results show that enhancement of OAS1 activation by the 3′-ssPy motif has preference for a C3′-endo sugar pucker and a pyrimidine base but is insensitive to the specific base identity, indicating that the Watson–Crick base edge is not a key determinant of recognition by OAS1. These observations suggested to us that strong shape complementarity between the 3′-ssPy motif and the adjacent OAS1 surface might be most critical, with recognition driven primarily by base stacking and/or packing of the ribose against the protein surface. We therefore sought to identify OAS1 residues to target for mutagenesis with the goal of revealing protein surface changes that render OAS1 insensitive to the 3′-ssPy motif, while maintaining its response to dsRNA.

**Figure 6.**
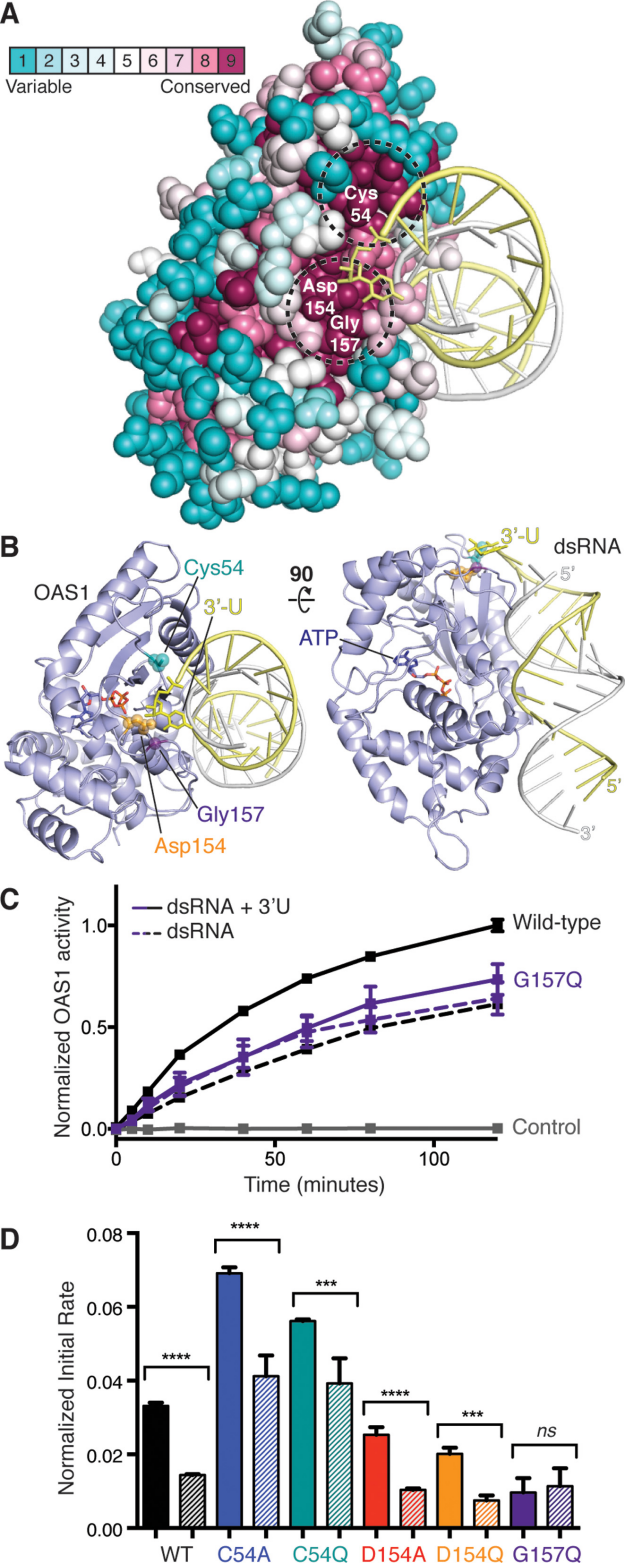
OAS1 G157 is a critical mediator of 3′-ssPy motif action. (**A**) Consurf analysis of OAS1 run using the X-ray crystal structure of the human protein (PDB ID: 4IG8) determined in complex with the 18-bp dsRNA duplex. Two highly conserved patches (dashed circles) are located adjacent to the approximate position of the modeled 3′-ssPy motif. (**B**) Two orthogonal views of the OAS1-dsRNA structure with mutated protein residues and modeled 3′-ssPy motif highlighted. (**C**) Chromogenic assay showing the effect of the 3′-ssPy on wild-type and G157Q mutant OAS1 activity. (**D**) Comparison of initial rate of reaction for wild-type and mutant OAS1 proteins activated by the 18-bp dsRNA duplex with (solid bar) or without (striped bar) an additional 3′-end single-stranded uridine residue (3′-ssPy motif). One-way ANOVA: *P* ≤ 0.0001 (****), *P* between 0.0001 and 0.001 (***) and not significant (ns; *P* ≥ 0.05). In panels (C–D), data are normalized to wild-type OAS1 activation by 18-bp dsRNA with the 3′-ssPy motif.

To identify candidate residues for mutational analysis, we used Consurf (35) to examine amino acid conservation among the 30 closest homologs of human OAS1. Two highly conserved regions on opposite sides of OAS1 were identified that correspond to the functionally critical dsRNA-binding surface and OAS1 catalytic center (Supplementary Figure S6). We modeled the likely location of the 3′-ssPy motif by manually appending an additional 3′-end uridine residue and thus identified two smaller conserved patches, residues C54 and D154/G157, in close proximity (Figure 6A and B). Each of these residues was individually mutated to specifically alter the protein surface, and thus potential shape complementarity with the 3′-ssPy motif, by incorporating bulkier (C54Q, D154Q or G157Q) and smaller side chains (C54A or D154A) at these locations.

We tested the ability of the 18-bp dsRNA duplex with and without the 3′ssPy motif to activate each mutant protein using the chromogenic assay and compared the initial rate of reaction under the conditions used (Figure 6C and D). Unexpectedly, we found that both mutations at residue C54 enhanced overall OAS1 activity, whereas mutations at D154 had no measureable effect. Critically, however, in each case no effect on the enhancement of OAS1 activation by the 3′-ssPy motif was observed (Figure 6D, compare solid and striped bars for each mutant). In contrast, G157Q reduced overall activation and in addition completely abrogated potentiation of OAS1 activation by the 3′-ssPy motif (Figure 6C and D). We conclude that G157 is critical and that the 3′-ssPy motif may exert its effect via direct contact with the protein surface containing this residue. That the effect of the 3′-ssPy motif may be governed by specific interaction with a short loop containing a single, highly conserved residue reveals a potentially powerful target for manipulation of OAS1 activity.

## DISCUSSION

Detection of the molecular hallmarks of invading pathogens and potent activation of the innate immune system are critical for survival of infection. The present work has revealed a novel molecular signature, the 3′-ssPy motif, required for optimal activation of OAS1 by both simple duplex RNAs and highly structured viral or cellular non-coding RNAs. Our results demonstrate that optimal activation of OAS1 by dsRNAs with a 3′-ssPy motif is critically dependent on the single-stranded nature of the motif with strong preference for pyrimidine base. While further studies are necessary to define the impact of the novel 3′-ssPy motif in a cellular context, our findings suggest a previously unappreciated mechanism by which the sensitivity of the OAS1/RNase L pathway is fine-tuned. Once activated by the products of OAS1, RNase L degrades single-stranded RNAs and single-stranded regions present within loops or bulges in structured RNAs, preferentially following UA or UU dinucleotide sequences (36). RNase L action thus increases the pool of dsRNA with a 3′-ssPy motif and may sensitize the cell to viral invaders through rapid amplification of OAS1 activity and RNase L-mediated RNA degradation. 3′-ssPy motif action may also underpin the RNase L-dependent amplification observed by others (37). Thus, the present work provides a valuable starting point for future cell-based experiments to define the role of the 3′-ssPy motif in infection.

Our analysis of OAS1 activity promoted by the simple 18-bp dsRNA or the structured non-coding adenoviral transcript VA RNA_I_ revealed a significant difference in the effect of the 3′-ssPy motif on apparent OAS1-RNA affinity (*K*_app_). The 18-bp dsRNA duplex is optimal in length to span the dsRNA binding surface of OAS1 and also contains an activation consensus sequence that likely directs the specific orientation adopted by this RNA (14). Our results show that, in this minimal context, the 3′-ssPy motif does not alter affinity but nonetheless directly promotes enzyme turnover (increased *V*_max_). Such enhancement could arise through a number of mechanisms, but we hypothesize that the 3′-ssPy motif enhances promotion of the necessary dsRNA-driven conformational change for catalysis by OAS1 or directly stabilizes the reorganized active structure. This scenario would further fine-tune this system, making OAS1 a powerful sensor of the continued presence of cytoplasmic dsRNA. In contrast to the simple dsRNA duplex, for VA RNA_I_ the 3′-ssPy motif significantly decreased RNA affinity (*K*_app_) while increasing OAS1 enzyme activity. RNA binding affinity and potency of OAS1 activation do not necessarily correlate (16). Thus, our observation of the effect of 3′-ssPy on OAS1 activation by VA RNA_I_ may reflect the fact that larger structured RNAs may possess multiple, potentially overlapping OAS1 sites, each with a distinct capacity to activate OAS1. The presence of the 3′-ssPy motif may direct OAS1 to the double-stranded terminal region of VA RNA_I_ over otherwise preferred binding sites with a lower potential for OAS1 activation. Further studies of OAS1–RNA interaction are needed to complete our understanding of OAS1 binding and activation by dsRNA. Such studies of structured RNAs may also be a useful tool in the search for additional RNA sequences or structures that can potently activate or inhibit OAS1.

**Figure 7.**
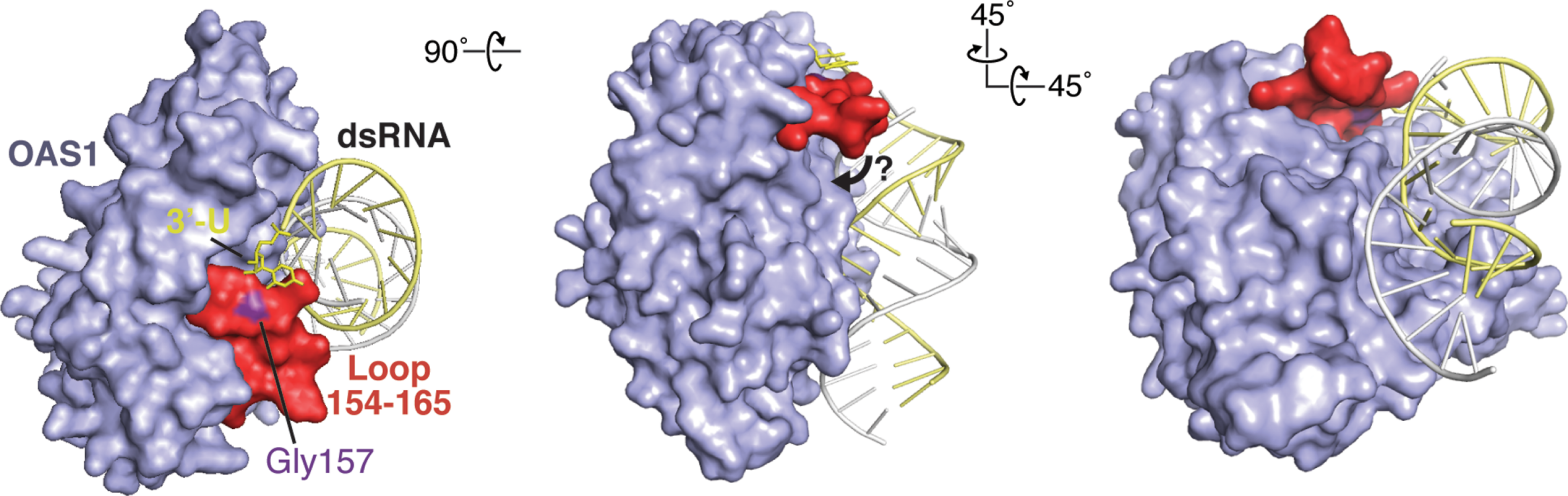
Model for 3′-ssPy motif action. Surface representations of the OAS1–dsRNA complex with modeled 3′-ssPy motif (3′-U). The OAS1 loop comprising residues 154 to 165 and the mutated residue that ablates dependence on the 3′-ssPy motif (Gly157) are highlighted. The three views are related by the rotations shown and the ‘left’ image corresponds to the orientation shown in Figure 6. A potential ‘closure’ of the 154–165 loop mediated by 3′-ssPy interaction at the surface comprising G157 is denoted with an arrow (center image).

Mutagenesis of OAS1 identified a single residue change, G157Q, that specifically ablated the effect of the 3′-ssPy motif. G157 is located in a short loop structure adjacent to the dsRNA binding surface that makes limited contact with the surrounding protein surface (14) (Figure 7). We suggest that the 3′-ssPy motif promotes interaction of this surface loop with other structural elements of OAS1 to promote the active state and that this loop may represent a potential novel target for therapeutic manipulation of OAS1 activity. OAS1 serum levels have been correlated with positive responses of Hepatitis C virus patients to interferon treatment (38), suggesting that further augmenting the OAS1 response may be beneficial. Additionally, activation of innate immune proteins such as OAS1 concomitant with chemotherapy could bolster patient response and increase survival rates (39).

Discovery of this novel signature for OAS1 activation presents the question of when, other than through viral replication or RNase L action, might dsRNA with the 3′-ssPy motif be present and accessible to OAS1? We found that the cellular nc886 RNA is a remarkably potent activator of OAS1. nc886 is proposed to prevent inadvertent PKR activation but to be displaced from PKR by viral RNA in the infected cell (32). Thus free nc886 may serve to sensitize the antiviral response through potent activation of OAS1. Further, the comparative potency of nc886 may reflect a role in OAS1/RNase L-mediated cell death in situations when infection has passed the ‘point of no return’. Additionally, aberrant accumulation in the cytoplasm of unprocessed RNA Pol III transcripts, Dicer products with 3′-overhangs or of miRNAs tagged for degradation by 3′-polyuridylation (40,41) could promote OAS1-mediated translational regulation and represent other contexts in which the 3′-ssPy might be important outside of infection. Thus, OAS1 activation may serve as a measure of a cell's suitability for active translation of proteins. In support of this concept is a recent and growing appreciation for OAS1-mediated translational control in cell cycle control, adipocyte differentiation and the role of RNase L in certain types of cancers (42–44). These diverse roles for OAS further highlight the importance of understanding the specific sequences or structural hallmarks that define an activating RNA as such.

## SUPPLEMENTARY DATA

Supplementary Data are available at NAR Online.

SUPPLEMENTARY DATA
