# Supplementary material for: A novel RNA molecular signature for activation of 2′-5′ oligoadenylate synthetase-1
Source: Nucleic Acids Res. 2014 Dec 4;43(1):544–52. doi: 10.1093/nar/gku1289 (PMC4288181; doi:10.1093/nar/gku1289)
Supplement: SUPPLEMENTARY DATA [file supp_43_1_544__index.html]

A novel RNA molecular signature for activation of 2′-5′ oligoadenylate synthetase-1 — SUPPLEMENTARY DATA 

# A novel RNA molecular signature for activation of 2′-5′ oligoadenylate synthetase-1

## SUPPLEMENTARY DATA

**Files in this Data Supplement:**

- SUPPLEMENTARY DATA
