# Supplementary material for: A novel RNA molecular signature for activation of 2′-5′ oligoadenylate synthetase-1
Source: Nucleic Acids Res. 2014 Dec 4;43(1):544–52. doi: 10.1093/nar/gku1289 (PMC4288181; doi:10.1093/nar/gku1289)
Supplement: SUPPLEMENTARY DATA [file supp_gku1289_Vachon-NAR-Supplementary-Data-corrected.pdf]

SUPPLEMENTARY DATA

A novel RNA molecular signature for activation of 2'-5' oligoadenylate synthetase-1

Virginia K. Vachon[1,2], Brenda M. Calderon[1,3] and Graeme L. Conn[1]*

[1]Department of Biochemistry, Emory University School of Medicine, Atlanta GA 30322, USA.

[2]Microbiology and Molecular Genetics (MMG) Program and [3]Biochemistry, Cell and Developmental Biology (BCDB) Program, Graduate Division of Biological and Biomedical Sciences, Emory University School of Medicine, Atlanta GA 30322, USA.

*To whom correspondence should be addressed. Tel:+1 (404) 727-5965; Fax: (404) 727-2738 ; Email: gconn@emory.edu

---

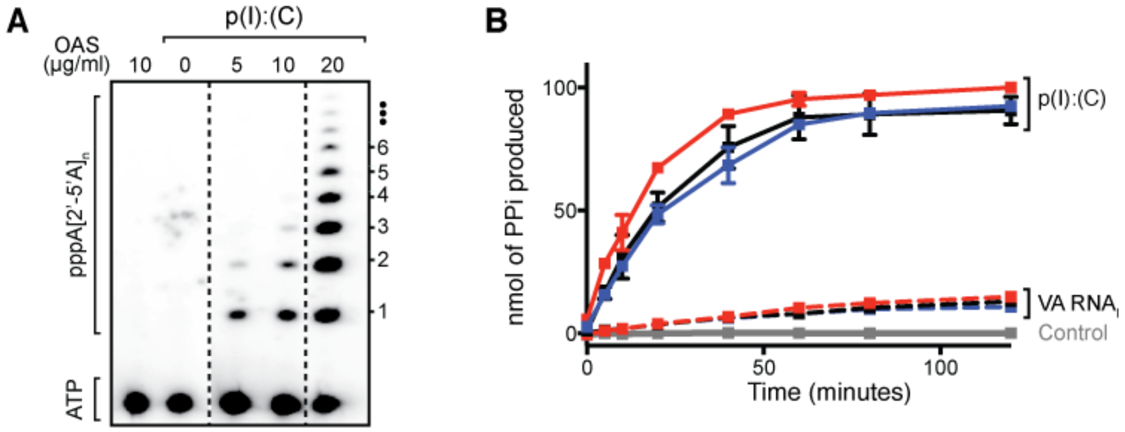

Supplementary Figure S1. Human OAS1 produced by cleavage of the SUMO-OAS1 fusion is active and shows only minimal prep-to-prep variation. *(A)* Phosphorimager analysis of denaturing PAGE demonstrating that OAS1 produced using the SUMO fusion system responds as expected to the known activator poly(I):poly(C) in the presence α-^{32}P-ATP. *(B)* As *panel A* but using chromogenic detection of PP_i produced as a by-product of oligoadenylate synthesis by OAS1 in response to poly(I):poly(C) RNA or full-length VA RNA_I. OAS1 prep-to-prep variability is minimal (compare red, blue and black lines for each RNA) and relative activation by each RNA for a given prep is maintained (compare solid and dashed lines).

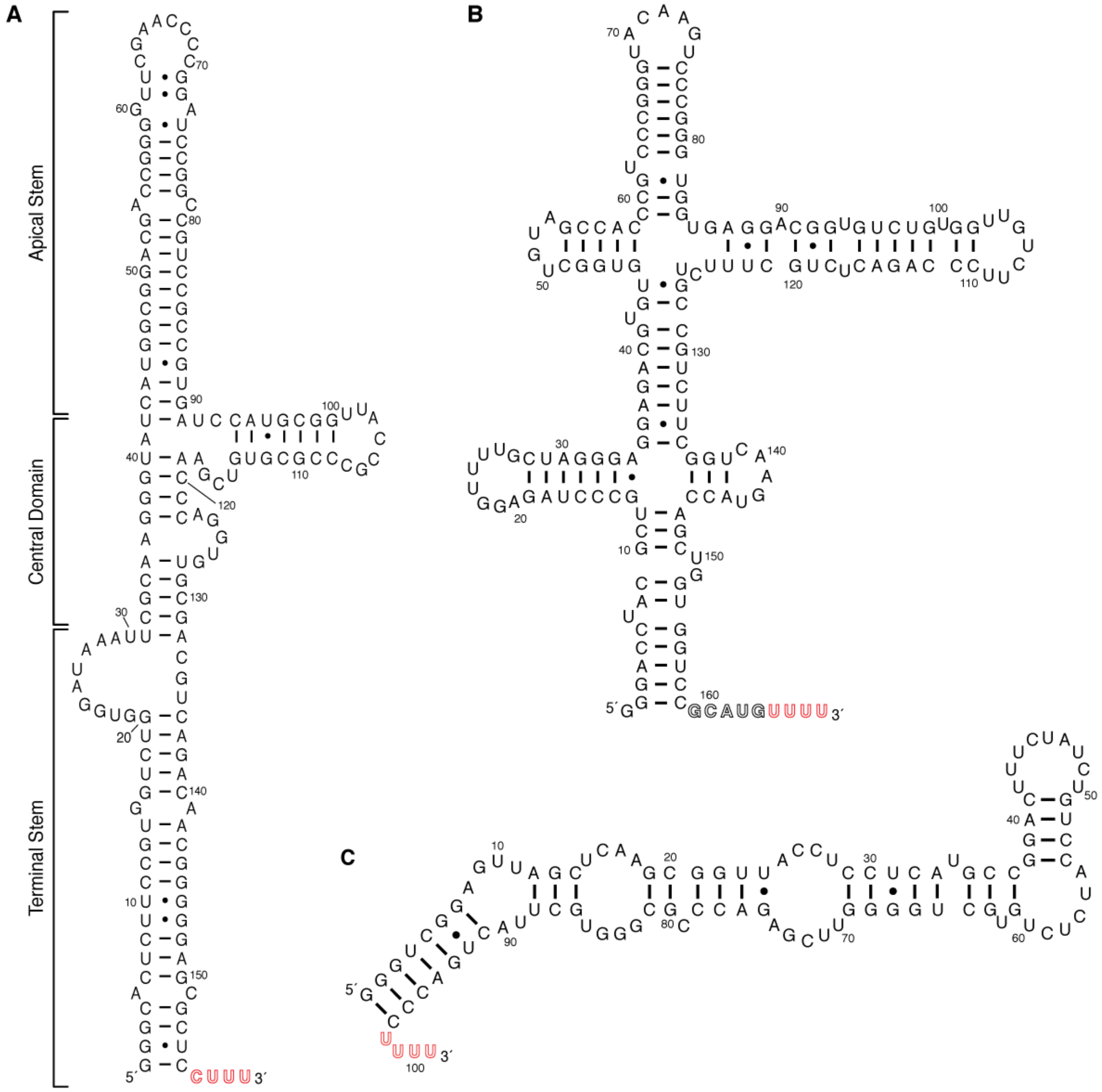

Supplementary Figure S2. Secondary and domain structures of the non-coding RNAs used in this study. *(A)* Adenovirus VA $\mathrm{RNA_I}$. *(B)* Epstein-Barr virus EBER-1 RNA. *(C)* Human cellular nc886 RNA. The short 3'-end single-stranded polyuridine sequence incorporated during RNA Pol III transcription termination highlighted (red outline font).

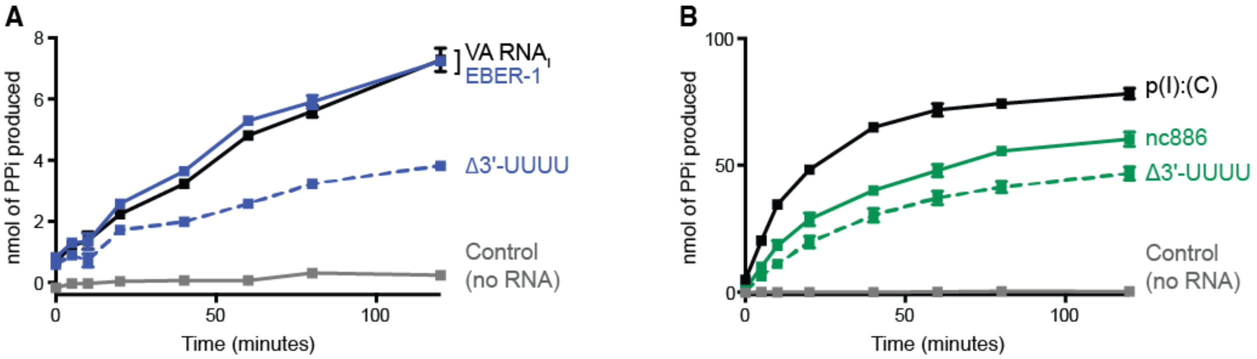

Supplementary Figure S3. Full activation of OAS1 by structured viral and cellular non-coding RNAs requires their 3'-end single-stranded pyrimidine-rich sequence (3'-ssPy motif). *(A)* Chromogenic assays showing optimal activation of OAS1 by EBER-1 RNA is dependent upon the presence of its 3'-end single stranded sequence. OAS1 activity is shown in the presence of wild-type EBER-1 (solid blue line), EBER-1 lacking the UUUU-3' sequence (Δ3'-UUUU; dashed blue line) and wild-type VA RNA$_I$ (black). *(B)* As *panel A* for the cellular nc886 RNA with and without the UUUU-3' sequence (green solid and dashed lines, respectively). Poly(I):poly(C) activator RNA (black) is shown for comparison as nc886 is an unusually potent activator of OAS1.

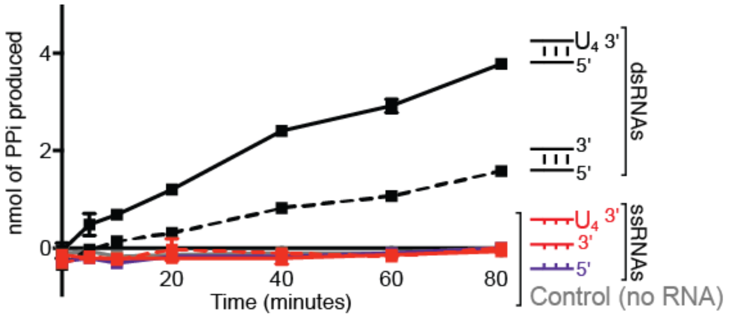

Supplementary Figure S4. ssRNA sequences corresponding to the 18 bp dsRNA do not activate OAS1. Chromogenic assays are shown of OAS1 activation by each forward and reverse ssRNA (0.3 μM; red and purple, respectively) or dsRNA duplex (0.3 μM) with and without the 3'-ssPy motif (black solid and dashed lines, respectively). OAS1 activity is tightly controlled with no activity observed in the presence of ssRNAs. Activation by the equivalent dsRNA sequences is optimal with the additional 3'-end single-stranded sequence. These data demonstrate that the 3'-ssPy motif must be part of a dsRNA in order to exert its effect.

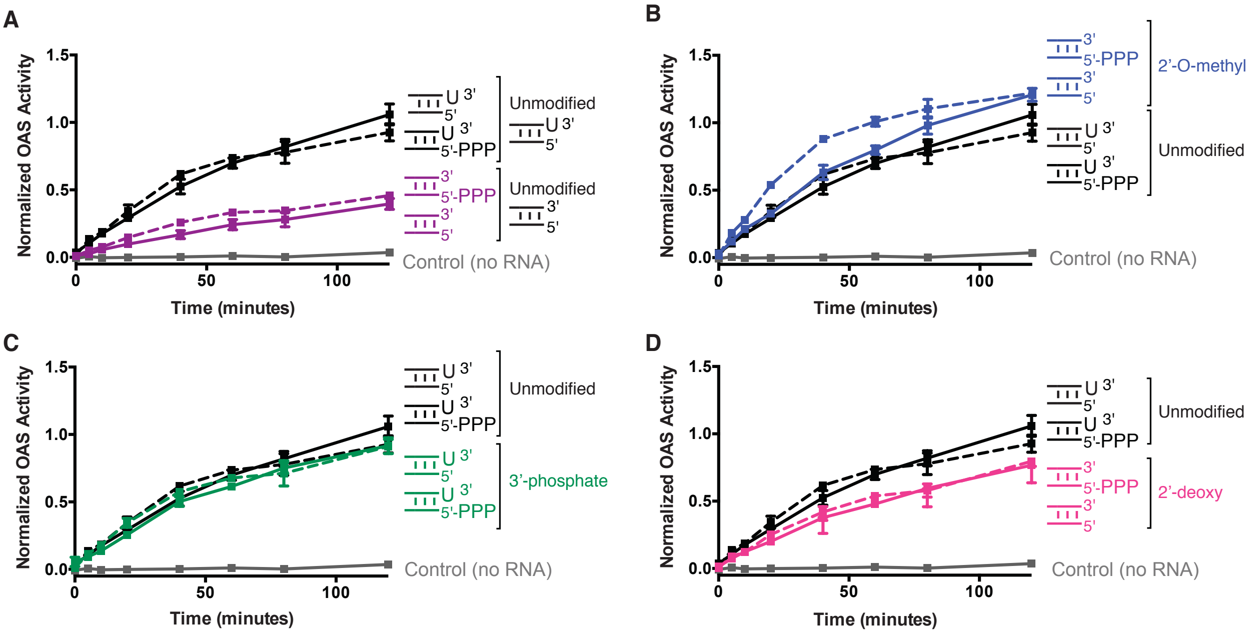

Supplementary Figure S5. A 5'-triphosphate group on the reverse strand of the model 18 bp dsRNA has little effect on 3'-ssPy motif activity. *(A)* Chromogenic assays of OAS1 activation by the 18 bp dsRNA without (purple) and with (black) a 3'-ssPy motif, in the absence (solid lines) or presence (dashed lines) of a 5'-end triphosphate (5'-PPP) on the complementary strand. Data in this and other panels are normalized to the final PPi produced in the presence of the 18 bp dsRNA with a 3'-ssPy motif but without a 5'-triphosphate on the complementary strand (solid black line). *(B-D)* The effect of the complementary strand 5'-triphosphate group on dsRNA duplexes with chemical modifications to the 3'-ssPy motif ribose group: *(B)* 2'-O-methyl (blue), *(C)* 3'-phosphate (green), and *(D)* 2'-deoxyribose (magenta). Reaction curves for the 18 bp dsRNA with an unmodified 3'-ssPy, with and without a 5'-triphosphate on the complementary strand (dashed and solid black lines, respectively), shown for comparison in each panel are the same as in *panel A*. The ability of the 3'-ssPy motif with chemical modifications to activate OAS1 was unaltered in the presence of a 5'-triphosphate group on the complementary strand, except for the 2'-O-methyl ribose modification for the initial rate of PPi production was modestly enhanced (compare dashed and solid blue lines in *panel B*). The data presented in this figure are the same as those shown with a different organization in **Figure 5** in the main text.

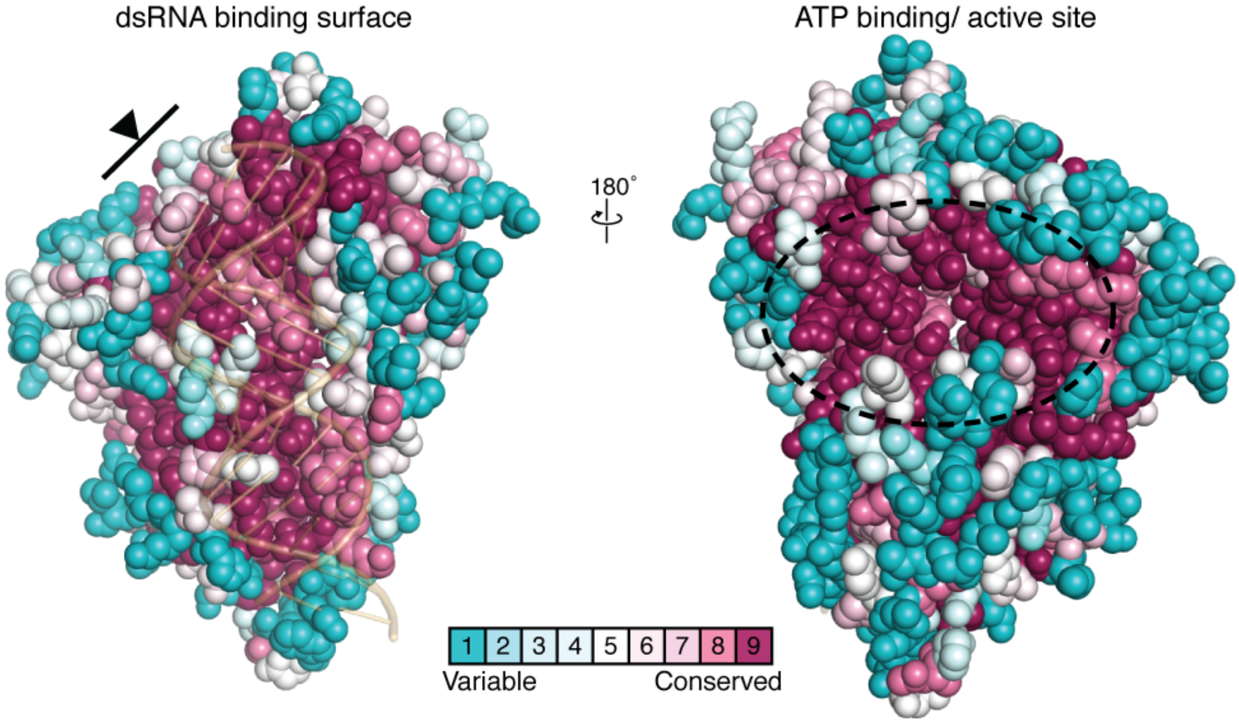

Supplementary Figure S6. **Consurf analysis of OAS1.** Consurf analysis (35) mapped onto the X-ray crystal structure of human OAS1 (PDB ID: 4IG8) determined in complex with the 18 bp dsRNA duplex (shown as transparent orange strands, *left*) highlighting the highly conserved dsRNA binding region (*left*) and ATP binding pocket (*right*, dashed circle). Regions predicted to interact with 3'-ssPy are located adjacent to the dsRNA binding surface (the plane and black arrow depict the orientation shown in **Figure 6**).
